# Noninvasive optical monitoring of pulmonary embolism: a Monte Carlo study on visible Chinese human thoracic tissues

**DOI:** 10.1117/1.JBO.28.1.015001

**Published:** 2023-01-18

**Authors:** Shuo Meng, Hengjie Su, Jianghui Guo, Lingxiao Wang, Ting Li

**Affiliations:** a Chinese Academy of Medical Sciences, Institute of Biomedical Engineering, Peking Union Medical College, Tianjin, China; b Tiangong University, Tianjin, China; c University of Electronic Science and Technology of China, Chengdu, China; d Chinese Institute for Brain Research, Beijing, China

**Keywords:** pulmonary embolism, near-infrared spectroscopy, Monte Carlo simulation, light fluence, signal sensitivity distribution

## Abstract

**Significance:**

In recent years, the incidence rate of pulmonary embolism (PE) has increased dramatically. Currently, the correct diagnosis rate of PE in China is relatively low, and the diagnosis error rate and missed diagnosis rate were as high as about 80%. The most standard method of PE detection is pulmonary artery digital subtraction angiography (DSA), but pulmonary artery DSA is an invasive examination, and patients can have certain risks and discomfort. Noninvasive monitoring of PE remains challenging in cardiovascular medicine.

**Aim:**

We attempt to study the light propagation in human thoracic tissues and explore the possibility of near-infrared spectroscopy (NIRS) in noninvasive detection of PE.

**Approach:**

In this study, by utilizing the Monte Carlo simulation method for voxelized media and the Visible Chinese Human dataset, we quantified and visualized the photon migration in human thoracic region. The influence of the development (three levels) of PE on the light migration was observed.

**Results:**

Results showed that around 4.6% light fluence was absorbed by the pulmonary tissue. The maximum signal sensitivity distribution reached 0.073% at the 2.8- to 3.1-cm light source–detector separation. The normalized light intensity was significantly different among different PE levels and formed a linear relationship ($r^{2}=0.998$, $p<10^{-5}$).

**Conclusions:**

The study found that photons could reach the pulmonary artery tissue, the light intensity was linearly related to the degrees of embolism, PE could be quantitatively diagnosed by NIRS. Meanwhile, the optimized distance in between the light source and detector, 2.8 to 3.1 cm, was recommended to be used in future potential noninvasive optical diagnosis of PE.

## 1 Introduction

Pulmonary embolism (PE) refers to a clinical and pathophysiological syndrome that commonly occurs as a cardiovascular disease.^1^ It shows pulmonary circulation disorders and even pulmonary infarction that are caused by endogenous or exogenous emboli block of the pulmonary artery or its branches. According to a report,^2^ the incidence of PE was the second only to coronary heart disease and hypertension in cardiovascular diseases, which is equivalent to 1/2 of the incidence of acute myocardial infarction; the mortality rate ranks third, the clinically observed acute PE mortality rate is not lower than that of myocardial infarction.^3^ The most accurate PE detection method is the pulmonary artery digital subtraction angiography (DSA), which is the gold standard for the PE diagnosis with almost 100% accuracy. However, this examination method is invasive and easy to cause damage to blood vessels or surrounding tissues. Besides, complications frequently occur after examination that also limit the DSA application.^4^ Another frequently used clinical noninvasive detection is the computer tomography pulmonary angiography (CTPA), which can detect pulmonary artery emboli through morphological character analysis, thus providing a basis for the PE diagnosis. However, due to the complex vascular structure below the pulmonary artery subsegment and the large interference outside the slice, the diagnostic efficiency of this part of embolus is reduced. Therefore, noninvasive detection of PE remains a challenge in cardiovascular medicine.

Near-infrared spectroscopy (NIRS) is based on the spectral characteristics of oxygenated hemoglobin (HbO2) and deoxygenated hemoglobin (HbO)^5^ and quantitative determination of tissue hemoglobin oxygen saturation for measure disease. Since its invention in the 1970s, with its advantages of noncontact, noninvasive, and online rapid detection, it has been used in a series of clinical human body studies with validated results.^6^ In recent years, great progresses in NIRS research were on human brain,^7^^,^^8^ heart,^9^ lung,^10^^,^^11^ and mammary gland.^12^ Accurate tissue modeling of tissue increases the utility of photon migration for a broader range of applications. NIRS can detect up to a few centimeters that are enough to penetrate the thorax and reach the pulmonary artery.^13^ However, the study of photon migration in human pulmonary artery region has not been thoroughly studied, and noninvasive optical technology has great prospects in clinical PE detection.

In previous studies, hemodynamic abnormalities occurred first with PE, followed by characteristic events associated with hypoxemia. It was evident that the right ventricular blood reperfusion into the pulmonary artery was in disorder under the instance of pulmonary artery lesions (e.g., increased levels of embolism). The changes of hemodynamic provided physiological basis for direct detection of PE under hypoxemia.^14^ At present, the migration rules of NIR light in the pulmonary artery are not clear, so it is impossible to determine where to place the light source and detector (LSD) and how much the separation of the LSD can better detect the signal sensitivity distribution (SSD). This is also an urgent problem to be solved to achieve the clinical application of noninvasive PE detection.

It is very important to obtain the quantitative characteristics of photon migration in the thoracic tissues model under noninvasive detection mode and to study the optimal design of light source detector placement. This study aimed to solve these problems. Before to know whether PE could be detected by the optical method, the photon transmission rules in pulmonary tissues were needed to understand. The photon transmission of human body was complicated. To be more clarified, the pulmonary artery study was simulated on a three-dimensional (3D) structure, VCH is the best choice. The VCH is an individualized 3D heterostructure model,^15^ which is suitable for the simulation of 3D voxel media. The VCH has played an important role in many studies.^16^^–^^18^ The Monte Carlo code MCML (Monte Carlo modeling of light transport in multilayered tissue) has been the gold standard for simulations of light transport in multilayer tissue,^15^^,^^19^ but it is ineffective in the presence of 3D heterogeneity. In fact, the team has developed a software program targeted for Monte Carlo simulation of light propagation in voxelized media (MCVM).^16^ Using the MCVM, we can better evaluate the relationship between photon propagation in VCH chest model and the layout between LSDs. At present, MCVM + VCH has been successfully used for studying the photon transmission distribution of brain, mammary gland, and heart, which showed the reliability of the MCVM+VCH method. However, this method has not been used for obtaining the photon migration characteristics of pulmonary artery yet.

In this study, thoracic tissues model of VCH dataset was adopted to improve the precision of chest structure modeling, and Monte Carlo simulation was carried out in combination with 3D voxelized media to establish a quantified and visualized photon migration human chest model. The SSD, the partial path-length factor (PPF),^15^ the differential path-length factor (DPF),^16^ and pulmonary artery signal contribution ratios of a series of separation of the LSD were calculated. The results showed that the degree of PE had a strong linear relationship with the detected signal intensity. We simulate and visualize photon migration in human thoracic tissues with MCVM + VCH and the potential of noninvasive PE detection was first studied.

## 2 Materials and Methods

### 2.1 Thoracic Model Built from the VCH

**Fig. 1:**
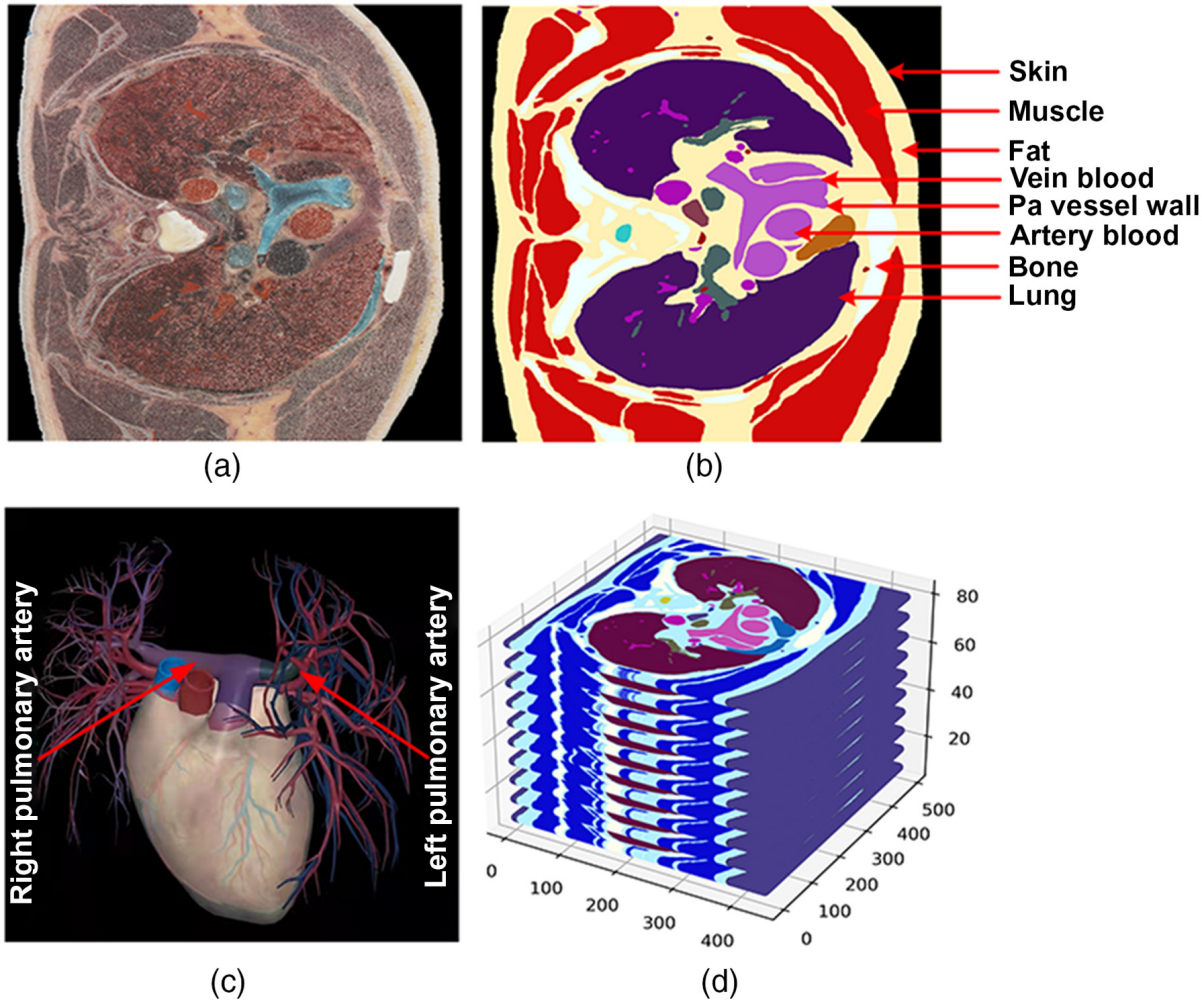
The description of VCH thoracic models. (a) A representative original thoracic photography image from VCH. (b) The thoracic model was divided into eight tissue parts. (c) A representative graph of the pulmonary artery structure (an open access picture from 3D Human Body app). (d) The thoracic model was built to the 3D matrix of 436×420×80  voxels.

The simulation was operated on a thoracic model established from the VCH dataset, which was a set of color photographs from the whole-body frozen sections. The VCH dataset was collected from a standing frozen Chinese male body (a donated cadaver) with little pathological alternations. In this study, we used 80 slices of photographic images of the VCH sections to complete the thoracic tissues region of the pulmonary artery from top to bottom, a photographic thoracic model of 436×420×80  voxels was first built (Fig. 1). One voxel was defined as a $0.4\times 0.4\times 0.4\;{\mathrm{mm}}^{3}$ cubic. The whole model was divided into eight parts according to tissues that included skin, muscle, bone, subcutaneous fat, lung, Pa vessel wall, arteries, and venous blood. Please note that the voxels outside the thoracic tissues were assigned as air and did not participate in Monte Carlo simulation.

### 2.2 Monte Carlo Simulation

**Fig. 2:**
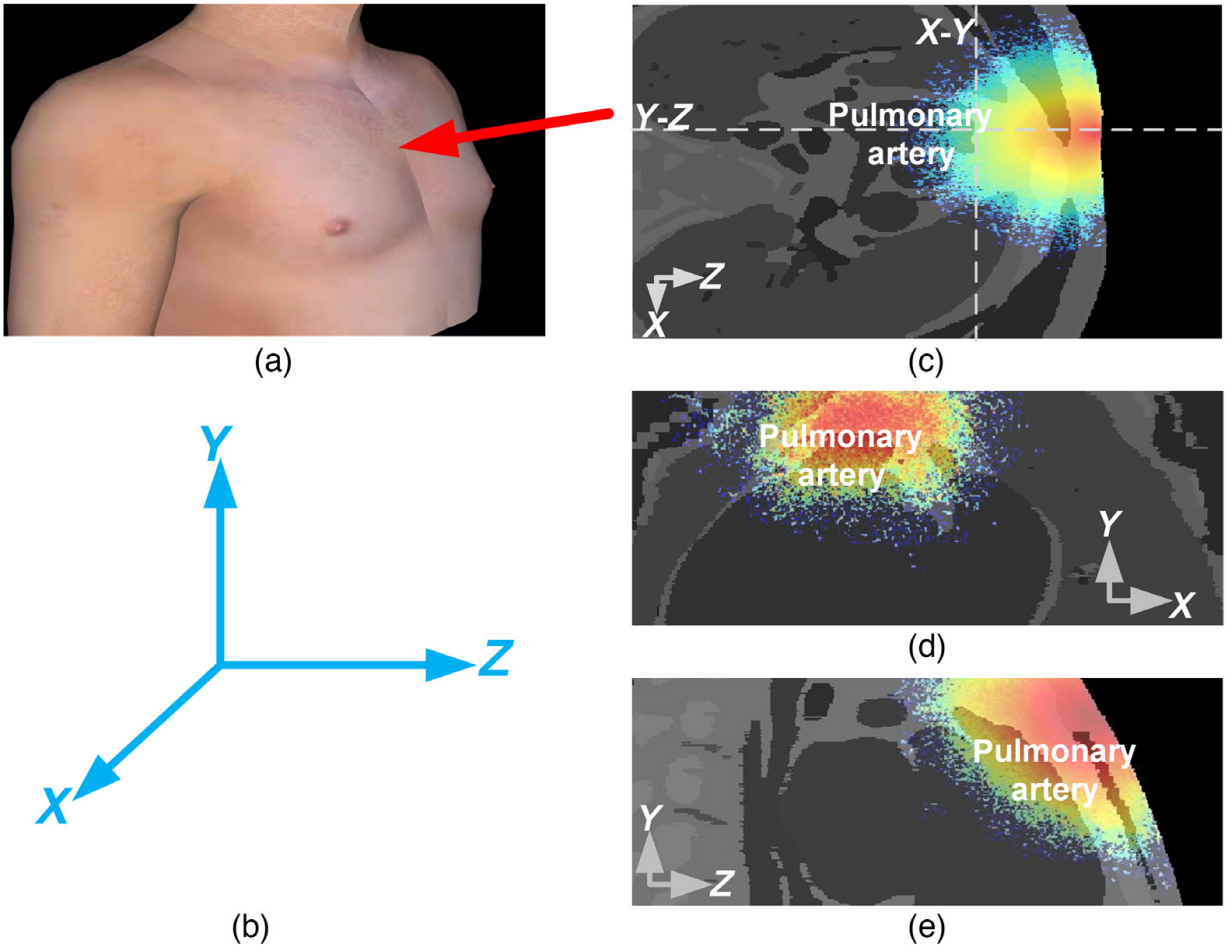
Light fluence distribution in thoracic tissue under X-Y-Z field of view. (a) The position of the LSD on the human thoracic model. (b) The three-dimensional space coordinate system used in this study. (c) Light fluence in the X-Z plane scale is 436×420. (d) Light fluence in the X-Y plane scale is 436×80. (e) Light fluence in the Y-Z plane scale is 420×80.

Monte Carlo simulation was used to deduce light propagation in the VCH of thoracic tissue. The Monte Carlo software used here was targeted for 3D voxelized media, the algorithm of which has been described in previous literature.^20^ In theory, when a photon packet (or a single photon) entered a tissue, it would be randomly scattered or continuously absorbed by the tissue media until it died.^21^ Survival photons collected by the detector could be analyzed to get the valued information, such as intensity and propagation path length. In this study, a light source was placed upon in between the second and third anterior ribs, the detector was attached to the skin on the horizontal left side of the light source, and the axis directions were shown in the Fig. 2. The input light was the 800-nm IR light and other set up parameters were the same with the previous studies and shown in the Table 1.^30^^–^^32^ Each simulation ejected $10^{6}\text{ photons}$. After 10 times simulation, the average photon fluence was calculated for representing the light propagation results. Besides, two files were generated for respectively recording the absorbed photon numbers and output photon distribution. According to these two files, a series of photon transmission characters were calculated.

**Table 1:** Optical properties of thoracic tissue at 800 nm.

Tissue type	n	$\mu_{a}$ (${\mathrm{cm}}^{-1}$)	$\mu_{s}$ (${\mathrm{cm}}^{-1}$)	g	References
Fat	1.33	0.083	134	0.910	22
Skin	1.37	0.20	73.7	0.715	23 and 24
Lung	1.40	1.0	95.0	0.910	25
Bone	1.43	0.11	291	0.936	26
Muscle	1.40	0.54	66.7	0.930	25
Pa vessel wall	1.40	0.92	103	0.930	27
Arterial blood	1.40	2.33	522	0.990	28 and 29
Venous blood	1.40	2.38	440	0.990	28 and 29

Where n is the refractive index of the medium, $\mu_{a}$ is the absorption coefficient, $\mu_{s}$ is the scattering coefficient, and g is the anisotropy factor.

Three disease levels were designed for simulating the optical transmission on the patients. We change the relationship between arterial blood and venous blood $\mu_{a}$ parameter to change the blood oxygen saturation.^33^ According to the variation of the arterial oxygen blood saturation (${\mathrm{SaO}}_{2}$) and the venous blood oxygen saturation (${\mathrm{SvO}}_{2}$), four groups were used in this study, including the three patient groups with mild (Level I), medium (Level II), and serious (Level III) disease, and the control group of normal people.^34^ The parameter details were shown in the Table 2.

**Table 2:** The parameters of $S_{A}$ and $S_{V}$ for the four groups.

Group	${\mathrm{SaO}}_{2}$ (%)	${\mathrm{SvO}}_{2}$ (%)
Level I	93	71
Level II	91	67
Level III	89	63
Normal	95	75

${\mathrm{SaO}}_{2}$ was the arterial blood oxygen saturation.

${\mathrm{SvO}}_{2}$ was the venous blood oxygen saturation.

### 2.3 Spatial Sensitivity Distribution

One output file of MCVM program contained the photon absorption in every voxel of the whole tissue model. The spatial distribution of light absorption ($A(\vec{r_{s}},\vec{r_{m}})$),^35^ a 3D matrix of 436×420×80  voxels, can be obtained from this file. Equation (1) was used to calculate the weight of the light fluence ($F(\vec{r_{s}},\vec{r_{m}})$) from the light source coordinate position ($\vec{r_{s}}$) to a random voxel (m) position ($\vec{r_{m}}$).^35^ The $\mu_{a}(\vec{r_{m}})$ was the absorption coefficient of the tissue type at the m’th voxel.^35^

Then ($\mathrm{SSD}(\vec{r_{S}},\vec{r_{D}},\vec{r_{m}})$) of the individual voxel was calculated by the three-point Green’s Eq. (2).^36^ $F(\vec{r_{D}},\vec{r_{m}})$ indicated the light fluence of a voxel ($\overset{\to }{r_{m}}$) at the ($\overset{\to }{r_{D}}$) location.^36^ After collecting all the SSD value of voxels, the SSD of the whole model was calculated by summing all these voxel SSD value.^36^ At the same time, the SSD of a specific tissue was calculated by summing all the voxel SSD value of this tissue.^37^ The ratio of the SSD of a specific tissue to total SSD represented the involvement of this tissue type in the light transportation^35^^–^^37^

$$F(\overset{\to }{r_{s}},\overset{\to }{r_{m}})=\frac{A(\overset{\to }{r_{s}},\overset{\to }{r_{m}})}{\mu_{a}(\overset{\to }{r_{m}})},$$ (1)

$$\mathrm{SSD}(\vec{r_{S}},\vec{r_{D}},\vec{r_{m}})=F(\vec{r_{S}},\vec{r_{m}})\times F(\vec{r_{D}},\vec{r_{m}}).$$ (2)

### 2.4 Separation of the LSD

The most optimal separation of LSD was calculated. As mentioned above, the light source was placed between the second and third anterior ribs, where had few muscles and could penetrate more photons. The detector was placed horizontally to the left of the light source. The separation of the LSD was determined by two factors, such as DPF and PPF.

DPF was the scaling factor that relates source-detector separations to the average path length that light travels. The notion of a DPF implies the assumption that the chromophore change was global to the sampling region.^38^ In contrast, PPF refers to a similar scaling factor but one that assumes a focal change. A PPF therefore represents a proportionality factor converting the source-detector separation into the average path length that light travels through a focal region of chromophore change.^39^^,^^40^

The PPF of the pulmonary artery (PPFpa) was calculated from the file that recorded the output photon distribution. It represented the sensitivity of the detected light intensity signal to the pulmonary artery.^26^ The PPFpa/DPF represented the contribution of pulmonary arterial tissue to the absolute absorption of photons. The optimal separation distance between the LSDs was reflected by such indicators of PPF and PPFpa/DPF.

## 3 Results

### 3.1 Light Fluence Distribution

As mentioned in the method part, light fluence distribution were drawn for indicating the photon existence in the thoracic tissues (Fig. 2). Penetrated photons could be observed reach to the pulmonary artery from any of the three views, such as X-Z, X-Y, and Y-Z. From the X-Z view, it was proved that the 800-nm light actually traveled through the sternum into the ribcage [Fig. 2(c)]. From the Y-Z light fluence distribution, it could be observed that photons scattered at the main pulmonary artery [Fig. 2(e)]. The X-Y distribution located at 33 mm toward the skin surface proved that photons could penetrate tissues to reach the pulmonary artery [Fig. 2(d)].

**Fig. 3:**
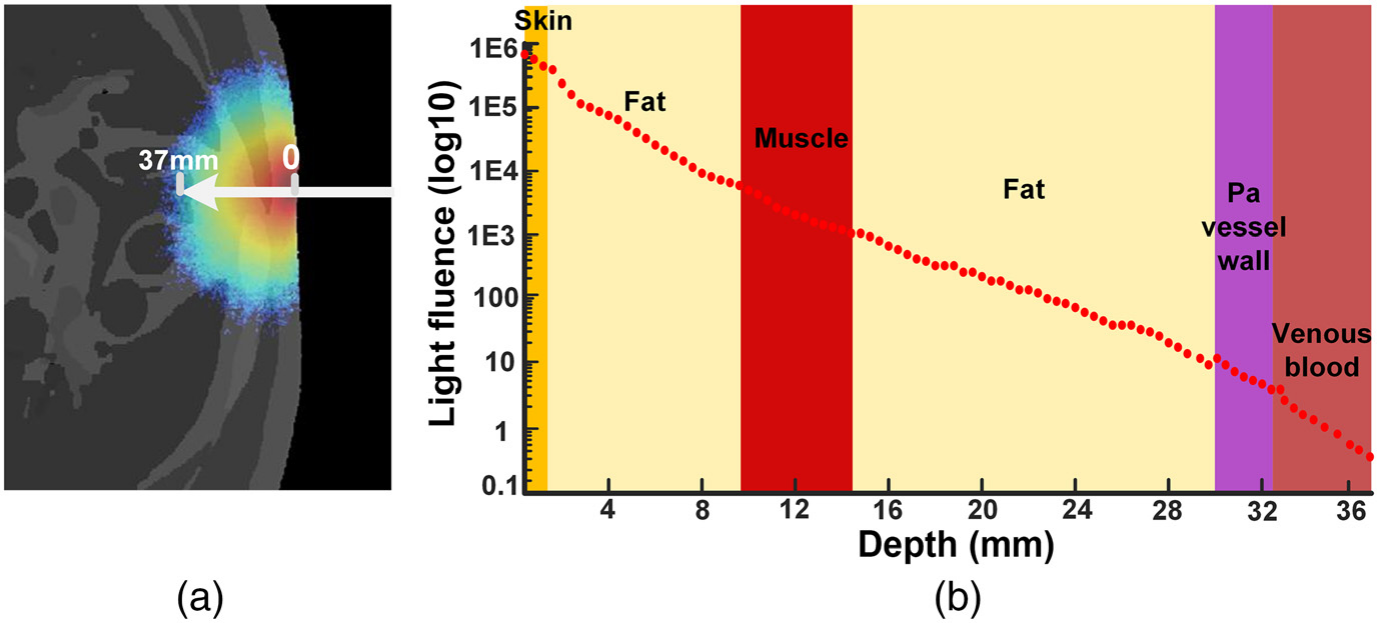
The light influence intensity changed with tissue depth. (a) The light fluence distribution on the 40th slice. (b) The light fluence intensity decreased from skin to venous blood.

A representative light fluence distribution on the 40th slice of X-Z view was shown in the [Fig. 3(a)]. It showed that the light intensity was set up to have photons traveling at least 37 mm in the body. The light influence intensity in log10 scale decreased as photons traveled through the tissues from skin, fat, muscle, fat, and pulmonary artery to venous blood [Fig. 3(b)]. Similarly, the light influence intensity decreased sharply in the normal scale.^41^ By a 3D coordinate system, set a center point position on the body surface (161, 333, and 40) and keep the X-axis and Y-axis unchanged. The photon attenuates from the incident point Z=333 until the light intensity is close to 0. The distance from the incident point to the end point is the penetration depth.^17^^,^^42^

When the depth increased to the pulmonary artery surface, the intensity was 45/voxel. At the depth of 36 mm, the intensity decreased to 1/voxel, at which venous blood was. These data suggest that 800-nm photon could transport into the pulmonary artery at depth of 36 mm with a light source placed upon anterior sternum. Furthermore, the light intensity decreased about $2.2\times 10^{4}$ times when the photons reached the pulmonary artery vessel wall.

### 3.2 Effect of Source–Detector Separation

**Fig. 4:**
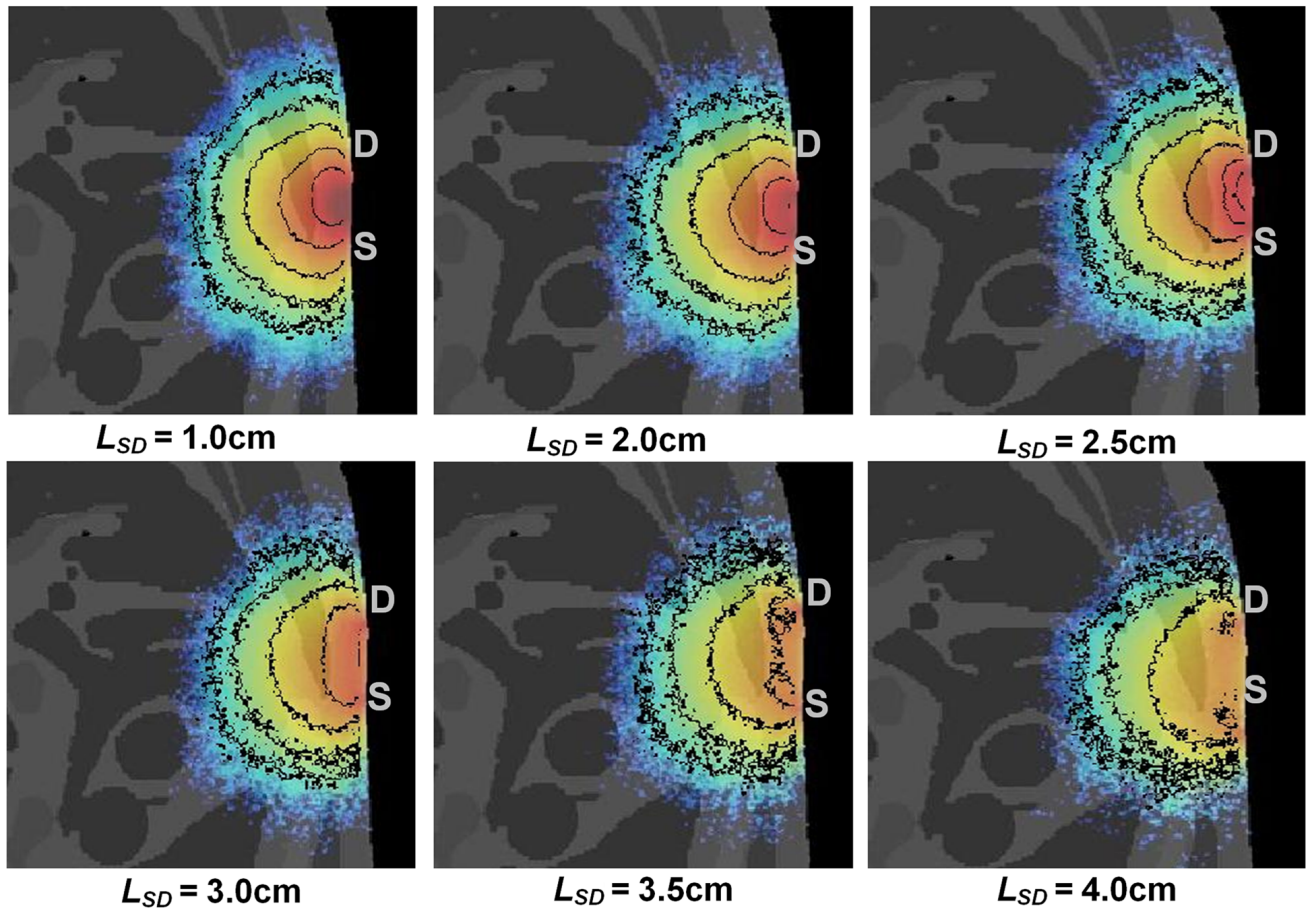
Spatial sensitivity profiles varied with $L_{\mathrm{SD}}$.

$L_{\mathrm{SD}}$ affected spatial sensitivity profiles.^43^ In this study, different $L_{\mathrm{SD}}$ was used for simulating the light propagation in the VCH thoracic lungs. The Fig. 4 showed the SSD on the 40th slice at various $L_{\mathrm{SD}}$. The results showed that the penetration depth and detection area of the spatial sensitivity profile gradually increased when the $L_{\mathrm{SD}}$ increased from 1.0 to 3.0 cm. When the $L_{\mathrm{SD}}$ increased from 3.0 to 4.0 cm, the detection area of the spatial sensitivity profile become broader but the penetration depth gradually decreased.

### 3.3 Optimized Source–Detector Separation

**Fig. 5:**
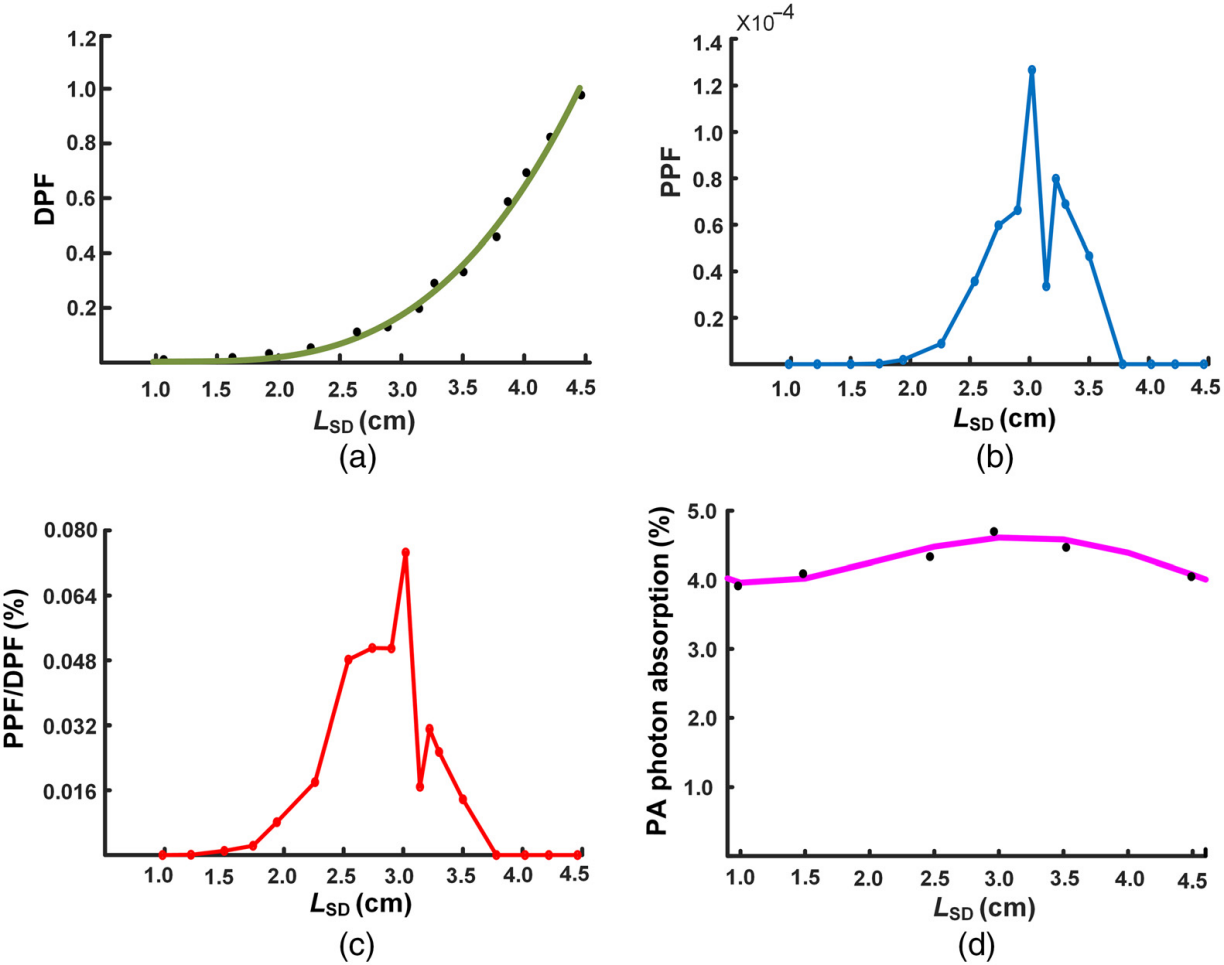
The value of DPF, PPFpa, PPFpa/DPF, and photon absorption at various $L_{\mathrm{SD}}$. (a) The DPF exponential growth. (b) The change of PPFpa at different $L_{\mathrm{SD}}$. (c) The change of PPFpa/DPF at different $L_{\mathrm{SD}}$. (d) The proportion of photon absorption at pulmonary artery.

The optimal separation of the LSD was depended on PPFpa, PPFpa/DPF, and pulmonary artery photon absorption. As shown in Fig. 5(a), DPFs tended to increase exponentially with the incremental $L_{\mathrm{SD}}$. Figure 5(b) showed that there was a different performance of PPFpa with $L_{\mathrm{SD}}$. When the $L_{\mathrm{SD}}$ was in the range of 2.5 to 2.9 cm, the PPFpa increased rapidly. And then, the PPFpa dropped sharply. Figure 5(c) showed that the change of PPFpa/DPF was affected by $L_{\mathrm{SD}}$. When the $L_{\mathrm{SD}}$ was in the range of 2.0 to 3.1 cm, the ratio of PPFpa/DPF increased gradually and then decreased sharply. There was a peak in between the 2.0 and 3.1 cm of $L_{\mathrm{SD}}$. By comparing the Figs. 5(b) and 5(c), both the PPFpa and PPFpa/DPF gradually increased to the maximum value when the $L_{\mathrm{SD}}$ increased from 2.0 to 2.8 cm and then sharply dropped when the $L_{\mathrm{SD}}$ increased from 2.8 to 3.1 cm. Figure 5(d) showed the absorption of photons by main pulmonary artery at different $L_{\mathrm{SD}}$. The absorption of photons rose slowly at initial and then decreased when the $L_{\mathrm{SD}}$ increased from 1 cm. The most optimal $L_{\mathrm{SD}}$ was about 2.8 to 3.1 cm where the photon adsorption reached the maximum of 4.6%. These results suggested that the optimized source-detector separation was in between the 2.8 to 3.1 cm.

### 3.4 Pulmonary Embolism Impact on SSD

The SSD also reflected the grade of PE.^44^ Table 3 showed the SSD proportion of each tissue at different PE levels. In the pulmonary artery, the SSD proportions were 0.0131%, 0.0129%, 0.0127%, and 0.0126%, respectively, for the healthy people, Level I patient, Level II patient, and Level III patient. When the PE becomes serious, the SSD proportion decreased. The SSD proportions of arterial and venous blood, as well as lung at different PE degrees were also estimated. It showed that the SSD proportion in lung and venous blood slowly decreased when the PE level increased. However, the SSD proportions in arterial blood were almost zero. These results indicated that the venous blood and lungs could be possibly used for detecting the PE by the SSD.

**Table 3:** The proportion of SSD (%) of each tissue at different levels of PE.

Tissue type	Fat	Skin	Lung	Bone	Muscle	Pa vessel wall	Arterial blood	Venous blood
Normal	67.398	11.34	0.002	15.92	5.34	0.0131	0	0.004
Level I	67.638	11.21	0.002	15.84	5.31	0.0129	0	0.004
Level II	67.588	11.27	0.002	15.89	5.25	0.0127	0	0.003
Level III	67.867	11.19	0.001	15.65	5.28	0.0126	0	0.002

### 3.5 Pulmonary Embolism Impact on Detected Light Intensity

**Fig. 6:**
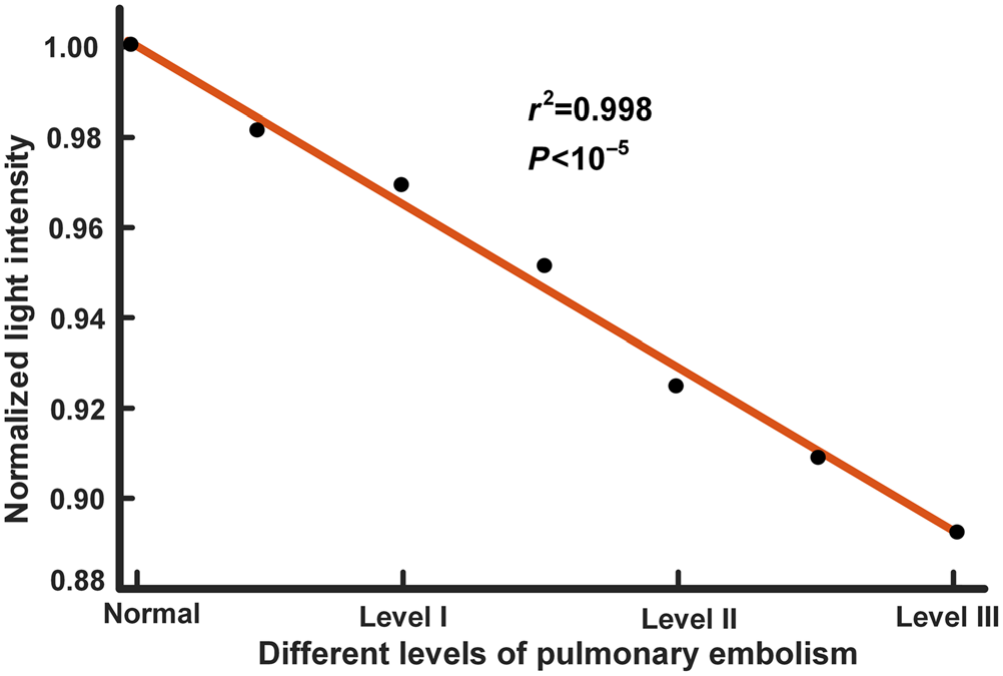
Linear fitting of normalized light intensity as the level of PE increased.

In the result of previous part, the optimal source–detector separation was around 2.9 cm. Hence, the $L_{\mathrm{SD}}$ was set to 2.9 cm for detecting the light intensity. In the result, light intensities were normalized to find the regular pattern (Fig. 6). Results showed that the normalized light intensity decreased as the PE level increased. Therefore, the light adsorption increased as the PE level increased. Though more photons were absorbed by the higher levels of PE, escaped photons were still detected by the detector. It proved that this detection method could be used for detecting the PE. The linear fitting function of normalized light intensity was ($r^{2}=0.998$, $p<10^{-5}$). This linear relationship suggested that the PE level could be accurately estimated by the detected light intensity.^45^

## 4 Discussion and Conclusion

In this paper, the Monte Carlo simulation based on the VCH was used for simulating the light migration in human thoracic tissues. Importantly, the number of absorbed photons and light fluence distribution in thoracic tissues were indicated partial photons could reach to the pulmonary artery. Besides, the different levels of PE impact on photon detection were confirmed by venous blood SSD and output photon intensity. In addition, the detected light intensity was linearly related to the PE level. These results suggested that PE could be accurately diagnosed by NIRS. In the end, the optimized $L_{\mathrm{SD}}$, 2.8 to 3.1 cm, was calculated by PPF, DPF, and the proportion of absorbed photons.

The optical parameters selected in this paper were obtained by the previous work of our group.^16^^,^^36^^,^^46^ These parameters were from studies of various groups,^22^^–^^24^^,^^27^ which were evaluated and confirmed by previous studies.^35^^,^^47^ In this study, the simulation experiment was carried out in an ideal condition, that was, the influence of the connection between organizations was ignored. Parameters that measured directly from the real human body were not suitable for this study. In the future work, more reality conditions will be included in simulation conditions. Then, the selected parameters will be changed. In Pifferi’s study, optical parameters of lung tissue were measured from the real human body, which included the influence of other tissue reaction.^10^^,^^11^ These parameters will be selected in the future work.

The NIR light has better performance in penetration depth.^48^ The pulmonary artery tissues get more light because of the low absorption coefficient of other tissues in NIRS, as presented in Table 1. In addition, the light source was placed on the skin upon the anterior sternum in this simulation. In the anterior sternum region, there were fewer muscles, and the distance between the skin and the pulmonary artery was relatively small, so the depth of the photon radiation into the pulmonary artery was greater. The photon distribution in the pulmonary artery tissues has been clearly displayed in Fig. 2. Moreover, the results suggest that certain photons may migrate to the surface of the body with optical information of the pulmonary artery tissues. After the incident light enters the human body, photons were absorbed and scattered in different tissues, and partial photons penetrate through the human skin, fat and bone then into the pulmonary artery.

The detector we use is a very common OPT101 (OPT101 Monolithic Photodiode and Single-Supply Transimpedance Amplifier).^49^ The sensitivity of this detector expressed by noise effective power is about $1.86\;(\mathrm{mA}/w)/{\mathrm{cm}}^{2}$ (800 nm), the size of photodiode is 2.29  mm*2.29  mm, and the size of OPT101 is 9.52  mm*6.52  mm. The OPT101 minimum detectable SSD signal is of order $10^{-6}$ to $10^{-7}$, so our measured SSD signal of 0.002% is still theoretically within the measurable range of existing devices. During simulation, we can set the receiving area of the detector to make it as consistent as possible with the detection area of the detector in reality. As shown in Table 3, pulmonary artery proportion of SSD was about 0.0131% of the total SSD, Venous blood only accounts for 0.002%. These values indicate that the photodetector can capture those photons that carry optical information about the target tissue and can analyze the captured signal.

Our results identified the optimized separation of the LSD depending on the DPF and PPF in Fig. 5. The largest diameter in the thoracic model can be narrowed down to 2.8 to 3.1 cm. In previous study, the optimal source-detector separation was narrowed down to 3 to 3.5 cm in the head model.^50^ PPFpa/DPF represents the contribution of the pulmonary artery to the absolute absorption measurement, and higher proportion value might be conducive to detection of absolute concentrations of oxyhemoglobin and deoxyhemoglobin to account for hemodynamics at the pulmonary artery. Actually, the separation distance between the light source and the detector was not as farther as better, when the distance was larger than 3.1 cm, the energy density of the emitted photon decreased rapidly, that seriously affected the detected signal quality.^51^

The PE was simulated in this study by produced different degrees of hypoxemia to simulate different levels of embolism at the pulmonary artery. The results showed that there were significant differences in optical performance of different PE levels in Fig. 6. The drop of light intensity reflected the embolism in the blood. The region beneath coverage of the NIRS probe contributes more sensitivity to NIRS measurements, and scattered photons outside this region can also be detected but with less reliability and sensitivity. This is a common problem with NIRS technology. Furthermore, as mentioned above, the light intensity variations could be a comprehensive outcome of various factors in the detected area, and light intensity as a single contrast agent sometimes may possibly issue a false alarm for embolism. The combination of multivariate modeling such as oxygen consumption and optical properties may make the prediction of PE more reliable. Nevertheless, to the best of our knowledge, this study was the first to study PE using an optical noninvasive approach.

From the simulation result, the light intensity could be an indicator of PE determination. In this study, the simulation experiment was carried out under ideal conditions, including the probe contacting the skin surface with zero distance and the probe not causing any pressure to the skin. The thickness and optical properties of the skin did not change. However, in human experiments, the light intensity is influenced by many factors, such as the optical contact distance between probe and tissue, optical properties and thickness of the skin, and the pressure of the probe to the tissue. Based on the simulation results, more real conditions will be considered in the future work of simulation and instrument design. Lightweight detectors might be used for constructing the instrument to reduce the pressure between the probe and the skin and reduce the influence to the detected light intensity. In addition, the tissue optical properties also change with the human exhalation and inhalation. In this study, the average value of the light intensity at exhalation and inhalation status was calculated to eliminate this change influence that represented a relative ideal condition. More complicated reality conditions of exhalation and inhalation will be considered in the future.

In conclusion, the present study employed the thoracic model of visible Chinese human and Monte Carlo simulation to investigate noninvasive detected of the PE levels. The study found that photons could reach the pulmonary artery tissue and the light intensity was linearly related to the degrees of embolism. The appearance of PE can produce lower intensity, which could be used as a reflection on the presence of embolus in pulmonary artery. Overall, this study demonstrated the great potential of NIRS in detecting PE.
